# Incidence and Prevalence of Early Onset Dementia in Finland

**DOI:** 10.1002/alz.085851

**Published:** 2025-01-09

**Authors:** Eino Solje, Mikko Aaltonen, Kalle Aho, Sami Heikkinen, Ave Kivisild, Adolfina Lehtonen, Laura Leppänen, Iina Rinnankoski, Helmi Soppela, Laura Tervonen, Annakaisa Haapasalo, Anne Remes, Päivi Hartikainen, Kasper Katisko, Johanna Krüger

**Affiliations:** ^1^ Kuopio University Hospital, Kuopio, Kuopio Finland; ^2^ University of Eastern Finland, Kuopio, Kuopio Finland; ^3^ University of Eastern Finland, Joensuu, Joensuu Finland; ^4^ University of Oulu, Oulu, Oulu Finland; ^5^ Oulu University Hospital, Oulu, Oulu Finland; ^6^ University of Helsinki, Helsinki, Helsinki Finland

## Abstract

**Background:**

Early onset dementia (EOD) forms distinct economic, societal, and human burdens on patients, their families, the healthcare system, and society, differing from those associated with late onset dementia. However, epidemiological data on EOD, characterized by onset before the age of 65, is notably scarce.

**Method:**

Our objective was to assess the incidence of all‐cause progressive Early Onset Dementia (EOD) and its subtypes from January 2010 to December 31, 2021 in two specified regions in Finland (Northern Ostrobothnia and Northern Savonia). All visits at the dementia outpatient clinics were manually reviewed during that period. In addition, prevalence of EOD and its subtypes were calculated on December 31, 2021 with 335,376 inhabitants in Northern Ostrobothnia and 186,592 in Northern Savonia.

**Result:**

In the population aged 30‐64 years, crude incidence was 20.5/100 000 persons at risk/year based on 711 new cases from January 2010 to December 31, 2021. The prevalence of EOD was 128.9/100 000 in the age group of 30‐64 years. Amnestic Alzheimer’s disease (48.1%) and behavioural variant frontotemporal dementia (13.1%) were the most frequent subtypes.

**Conclusion:**

EOD represents a significant group of neurological disorders leading to early loss of work capacity, dependence of external assistance, and eventually to premature death. The outcomes of this study aim to assist authorities and healthcare professionals in organizing effective diagnostics and improved care tailored to the specific needs of EOD patients.

